# Leptin signalling, obesity and prostate cancer: molecular and clinical perspective on the old dilemma

**DOI:** 10.18632/oncotarget.5574

**Published:** 2015-09-10

**Authors:** Heba Alshaker, Keith Sacco, Albandri Alfraidi, Aun Muhammad, Mathias Winkler, Dmitri Pchejetski

**Affiliations:** ^1^ Department of Surgery and Cancer, Imperial College London, London, UK; ^2^ Department of Pharmacology and Biomedical Sciences, Faculty of Pharmacy and Medical Sciences, Petra University, Amman, Jordan; ^3^ University of Malta Medical School, Mater Dei Hospital, Tal-Qroqq, MSD, Malta; ^4^ School of Medicine, University of East Anglia, Norwich, UK

**Keywords:** obesity, BMI, prostate cancer, mortality, progression, adipokine, leptin, therapy

## Abstract

The prevalence of global obesity is increasing. Obesity is associated with general cancer-related morbidity and mortality and is a known risk factor for development of specific cancers. A recent large systematic review of 24 studies based on meta-analysis of 11,149 patients with prostate cancer showed a significant correlation between obesity and the risk of advanced prostate cancer. Further, a sustained reduction in BMI correlates with a decreased risk of developing aggressive disease. On the other hand, the correlation between consuming different products and prostate cancer occurrence/risk is limited.

Here, we review the role of adipose tissue from an endocrine perspective and outline the effect of adipokines on cancer metabolism, with particular focus on leptin. Leptin exerts its physiological and pathological effects through modification of intracellular signalling, most notably activating the Janus kinase (JAK) 2/signal transducer and activator of transcription (STAT) 3 pathway and recently shown sphingolipid pathway. Both high levels of leptin in circulation and leptin receptor mutation are associated with prostate cancer risk in human patients; however, the *in vivo* mechanistic evidence is less conclusive.

Given the complexity of metabolic cancer pathways, it is possible that leptin may have varying effects on prostate cancer at different stages of its development, a point that may be addressed by further epidemiological studies.

## OBESITY AND CANCER

There is currently a global epidemic of obesity. Overall, 33% of the world's adult population are overweight or obese, according to a survey conducted in 2005 [1]. If this trend continues, by 2030 this figure will have doubled. Epidemiological studies highlight obesity and its associated complications as a major health problem [2].

Obesity is a recognised risk factor for diabetes, arteriosclerosis, ischaemic heart disease and hypertension. Further, epidemiological studies have shown that obesity is associated with a multitude of cancer types including colorectal, hepatic, oesophageal, pancreatic, endometrial, ovarian and post-menopausal breast cancer [3-5]. Obese cancer patients have a higher death rate in comparison to their non-obese counterparts, with the increased death rate presenting for all cancers combined, regardless of whether obesity was a risk factor in the development of the specific cancer [3]. The pathophysiology of obesity-associated cancer varies with cancer type. Recognised mechanisms include chronic elevated insulin levels and insulin resistance, persistent local inflammation and higher secretion of steroid hormones (reviewed in [2]).

At present, there are reports of a robust empirical correlation between obesity and several types of cancer [3-5]. Due to the relative ease of obtaining patients' height and weight, especially important when conducting large-scale studies, in the vast majority of previous studies, obesity has been measured using body mass index (BMI), defined as the weight (kilograms) divided by the square of the height (meters). The epidemiologic evidence links an increased BMI to an increased propensity to oesophageal adenocarcinoma, thyroid, kidney, and colon cancer among men whereas strong correlations between BMI and endometrial, gallbladder, kidney, and oesophageal cancer have been established in women [6]. In both sexes increased BMI was associated with decreased survival of patients with cancer of the oesophagus, colon, liver, gallbladder, pancreas, and kidney, as well as non-Hodgkin's lymphoma and multiple myeloma [3, 5].

## OBESITY AND PROSTATE CANCER: CLINICAL EVIDENCE

Historically, there is a considerable amount of available data indicating a lack of association between prostate cancer incidence and high BMI (Table 1) [7, 8]. A large prospective study of BMI/weight change in relation to prostate cancer incidence and mortality found no correlation between prostate cancer incidence and an individual's high BMI. Nevertheless, higher BMI/weight gain is correlated with increased propensity to die from prostate cancer [8]. This apparent controversy may potentially be explained by the fact that testosterone (often suppressed in men with high BMI) is required for prostate tissue proliferation and therefore an inverse association between BMI and indolent prostate cancer is observed. On the other hand, a recent study has shown that testosterone helps maintain prostatic epithelium differentiation [9] indicating that low testosterone may increase the risk of developing poorly differentiated and hormone-insensitive prostate tumours. A recent report confirmed these findings of increased aggressiveness and mortality (but not prostate cancer incidence) in men with higher weight and BMI [7]. Further, obesity is associated with higher rate of Prostate specific antigen recurrence following external-beam radiotherapy or radical prostatectomy [10]. A biopsy cohort analysis showed that obesity was associated with a high-grade Gleason score on diagnosis of prostate cancer despite raised BMI not being a significant risk for developing prostate cancer [11]. Both BMI and waist circumference are predictors of high-grade prostate cancer, however obesity with central adiposity was shown to be the strongest predictor of diagnosing prostate cancer and high-grade disease [12]. A recent large trial of 2235 biopsy patients in Canada investigating the influence of the metabolic syndrome (any three of five components: obesity, elevated blood pressure, diabetes or impaired fasting glucose, low high-density lipoprotein-cholesterol, and hypertriglyceridemia) on Prostate cancer has shown that while no individual metabolic syndrome component was independently associated with Prostate cancer, overall metabolic syndrome was associated with higher Prostate cancer grade (p<0.001), as well as progressively higher odds of Prostate cancer outcomes: clinically significant prostate cancer and intermediate or high grade prostate cancer [13]. A recent study has shown that the presence of metabolic syndrome is a significant risk factor for shorter progression free survival in CRPC patients treated with abiraterone [14]. The association between obesity and aggressive prostate cancer is stronger in Caucasian Americans as opposed to African Americans; however, the latter tend to have more aggressive disease independent of obesity [15].

**Table 1:** Association of BMI with prostate cancer incidence

Study	Year	Sample Size	OR/HR/RR
Wright et al.	2007	287,760	RR 0.67
Su et al.	2011	1132	OR 1.48
Bassett et al.	2012	17,045	HR 1.06
De Nunzio et al.	2013	668	OR 1.05

This table highlights key articles that studied the association between a high baseline body mass index (BMI) and risk of subsequently developing prostate cancer. The association is expressed as an odds ratio (OR), hazard ratio (HR) or relative risk (RR). Su et al., studied the association of BMI with aggressive prostate cancer. Adapted from (Bassett et al., 2011; Su et al., 2011;De Nunzio et al., 2013; Wright et al., 2007).

With respect to decreased incidence of prostate cancer in relation to weight loss, men who maintained weight loss of greater than 11 pounds over a 10-year period had a decreased risk of non-metastatic high-grade prostate cancer. However, while high BMI was positively associated with non-metastatic high-grade prostate cancer it was simultaneously inversely associated with the risk of developing low-grade disease [16]. This may suggest that in contrast to low-grade prostate cancer, the pathogenesis of high-grade disease may be intrinsically linked to obesity-driven signalling. Indeed, meta-analysis of 16 published studies of 6569 cases and 8405 controls for the leptin receptor G2548A mutation showed that it is statistically significantly associated with an increased risk of prostate cancer (OR=1.26, 95% CI=1.05-1.51) [17]. Furthermore, men with high-volume prostate cancer (greater than 0.5cc in volume or extraprostatic disease) had higher serum leptin concentrations than their low volume counterparts when stratified by age, testosterone level, height and BMI [18].

In contrast to BMI, in a cohort study of 8152 men followed up for 15 years dietary carbohydrates (cake, biscuits, rice and pasta) have been associated with developing a low-grade prostate cancer, but not with high-grade disease [19]. A study on fatty acid intake in relation to prostate cancer risk identified an increased risk of advanced prostate cancer with increased dietary intake of α-linolenic acid, but it was not associated with risk of total prostate cancer [20]. However in a recent large systematic review and meta-analysis of 11,149 patients with prostate cancer the correlation between consuming different products and prostate cancer occurrence/risk was limited and inconclusive [21]. A recent single centre study showed that increased physical activity is associated with a reduced risk of prostate cancer and of high-grade prostate cancer on biopsy [22].

## OBESITY AND PROSTATE CANCER: CELL AND ANIMAL MODELS

Tumour growth in immunodeficient mice injected with prostate cancer LAPC-4 xenografts was slower in mice on low-fat diets as opposed to their high-fat diet counterparts [23]. Several proteins secreted by adipocytes play a role in prostate cancer progression. The expression of leptin receptor (LEPR) in prostate cancer influences the extent of tumour differentiation with no remarkable correlation between leptin levels in tumour tissues and BMI [24] or between circulating plasma leptin levels and BMI [25]. Collectively, the available evidence suggests that although it is unlikely for obesity to affect incidence of prostate cancer *per se*, obesity may affect the progression of existing prostate cancers. A role of adipokines in the aggressiveness of prostate cancer in obese males has been proposed [26]. This hypothesis states that cancerous prostate cells from obese individuals have a disrupted metabolic status, as they are being exposed to elevated levels of adipokines (e.g. leptin, interleukin (IL)-6 and vascular endothelial growth factor (VEGF), either *via* the circulation or in a localised manner upon the invasion of a retropubic fat pad. As a consequence of the abundance of obesity-related molecules, prostate cancer cell proliferation, differentiation, and angiogenesis can be promoted, worsening the pathophysiological outcome [26]. In addition to adipokines, excess body fat leads to altered serum levels of hormones, such as testosterone and insulin, which also might play a role in prostate cancer progression [27]. Murine prostate cancer cells exposed *in vitro* to obese sera had upregulated vimentin, β-catenin, e-cadherin dispersion and matrix metalloproteinase-9 which correlates with increased cellular invasion and migration. This may be a plausible mechanism in human cancer cells that correlates to prostate cancer aggressiveness [28]. There are recent reports showing a novel mechanisms of leptin signalling, linking it to the sphingolipid signalling [29, 30]. Sphingosine kinase 1 was shown to be highly active in human prostate tumours [31] and linked with prostate cancer chemoresistance [32-37].

## OBESITY AND ADIPOKINES

Obesity is defined by excessive growth of adipose tissue [2]. Two distinct types of adipose tissue have been identified: white adipose tissue (WAT) and brown adipose tissue (BAT). WAT constitutes the vast majority of the body's adipose tissue and functions as a storage site for excess fat in the form of lipid. On the other hand, BAT exists mostly in human neonates and regulates energy expenditure by adaptive thermogenesis. In addition to its storage role, the excessive growth of adipose tissue is detrimental to many physiological processes. Thus, the current paradigm of adipose tissue physiology has been revised based on its ability to act as an extremely active endocrine organ. In this context, WAT responds to and emanates bioactive substances that regulate biological processes like energy homeostasis, immunity and endocrine functions [38].

More than fifty adipose-derived factors, collectively termed adipokines, have been identified. Adipokines are proteins produced mainly by adipocytes, which comprise the majority of WAT. These secreted proteins comprise cytokines (e.g. tumour necrosis factor (TNF)-α and IL-6), angiogenic factors (e.g. VEGF and apelin) as well as other factors produced mainly by adipocytes (e.g. leptin and adiponectin) [38]. In addition, different parts of the body have distinctive adipokine profiles, rendering adipokines even more heterogeneous with respect to location. Overall, adipocytes have an extensive communication network of autocrine-paracrine signals with other tissues and organs, which has a significant impact on tissue homeostasis.

## LEPTIN SIGNALLING AND ITS PHYSIOLOGICAL ROLE

The discovery of leptin in 1994 shed light on one of the most important adipose tissue derived factors [39]. Leptin (from the Greek *leptos* meaning thin) is a versatile 16 kDa polypeptide encoded by the *obese* (*Ob*) gene (*LEP* gene by HUGO nomenclature) [39]. Leptin is mainly produced in adipose tissues, but is also synthesised in other, non-adipose tissue sites like the stomach, skeletal muscles and mammary epithelium.

Leptin acts as a circulating hormone to regulate appetite and the expenditure of energy *via* its action on specific receptors expressed in the hypothalamus [40]. The levels of leptin in plasma are proportional to fat mass and increase as body weight rises [40]. In humans, leptin circulates as a free hormone or bound to a soluble LEPR. The ratio of bound/free leptin varies between high levels in lean individuals, with low amounts of adipose tissue, and low levels in obese subjects, where the majority of leptin available is in its free form [41]. Additional pleiotropic effects of leptin include modulation of immune responses, angiogenesis, neovascularization and bone formation [42]. LEPR was first cloned from a mouse choroid plexus cDNA library [43]. LEPRs are products of a single *LEPR* gene. Further analysis of both human and mouse LEPR revealed the presence of multiple isoforms, including a form with a longer intracellular domain comprising approximately 306 amino acid (shorter in mice than in humans) hosting motifs, suggestive of intracellular signalling potential. Indeed, LEPR has structural similarities to members of the class-I cytokine receptor family. It shares highest sequence similarity with glycoprotein 130 (gp130) (a signal transducing component of IL-6 receptor), leukaemia inhibitory factor, oncostatin receptors, and with the receptor for granulocyte colony-stimulating factor. Therefore, LEPR possesses no intrinsic tyrosine kinase activity and initial signalling events are dependent on association with kinases like Janus kinase (JAK)2 [44].

The extracellular domain of LEPR is similar among the different isoforms due to the fact that differences in receptor isoforms arise from alternative RNA splicing at the C-terminus, resulting in different sequences and lengths of the intracellular domains. Six different isoforms of LEPR have been identified in mice [44] while four have been identified in humans. In all species, LEPR can be divided into three classes: long, short and soluble isoforms.

Both long and short isoforms of LEPR exist in dimeric form, even in the absence of leptin, and are activated upon leptin binding to the extracellular domain of LEPR in a 1:1 ratio. Dimerisation is pivotal for receptor activation and a prerequisite for execution of downstream signalling [45]. All isoforms (except the soluble isoform) share an identical intracellular domain, which associates with JAK, critical for JAK2 activation. The longest isoform of LEPR also contains a Box2 binding site and signal transducer and activator of transcription (STAT) binding sites [46]. The shortest isoform is soluble and is not involved in leptin signalling, as both the transmembrane and cytoplasmic domains are absent. However, it can act as a leptin-binding protein to regulate the circulating levels of free leptin [41].

**Figure 1:**
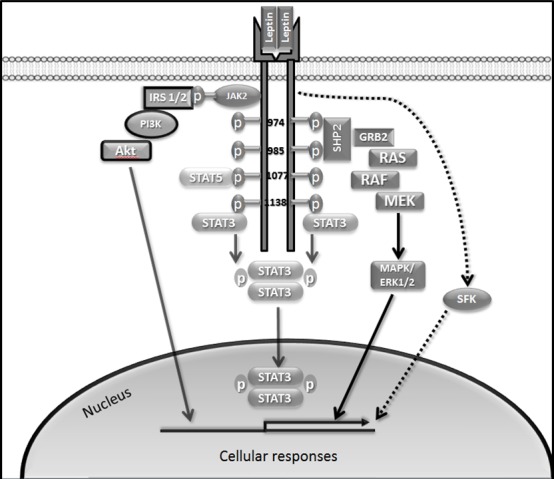
Role of LEPR-Long phosphorylation in leptin signalling LEPR-Long contains tyrosine residues (Tyr 974, Tyr 986, Tyr1077 and Tyr 1138). Tyr 1138 recruits STAT3 while Tyr 1138 and Tyr 1077 recruit STAT5. Phosphorylation at Tyr 986 and on Tyr 974 leads to SHP2 binding. JAK2 auto-phosphorylation at the Box1 motif leads to phosphorylation of IRS1/2, which can activate PI3K/Akt pathway. SFKs are also activated by leptin. Akt, protein kinase B; JAK2, Janus kinase 2; IRS1/2, insulin receptor substrate 1/2; GRB2, growth factor receptor-bound protein 2; MAPK, mitogen-activated protein kinase; PI3K, phosphatidylinositol 3 kinase; SHP2, SH2 domain-containing tyrosine phosphatase 2; STAT3, signal transducer and activator of transcription 3.

LEPR-Long hosts three conserved tyrosine residues linked to activation of distinct downstream signalling pathways (Tyr 986, Tyr 1077 and Tyr 1138) (Figure 1). Importantly, Tyr 1138 becomes phosphorylated following leptin binding. As a consequence, recruited STAT3, *via* its Src homology (SH)2 domain, becomes activated, followed by homodimerisation and nuclear translocation [47]. Tyr 1077 and Tyr 1138 can activate other STAT isoforms in addition to STAT3. Both Tyr 1077 and Tyr 1138 bind STAT5 whereas Tyr 1138 recruits STAT1.

Activation of Tyr 985 is required for triggering rat sarcoma (Ras)/rapidly accelerated fibrosarcoma (Raf)/extracellular signal-regulated kinases 1/2 (ERK1/2) pathways through interaction with an adaptor protein, growth factor receptor-bound protein 2 (GRB2) [47]. Phosphorylation at Tyr 986 (Tyr 985 in rodents) and Tyr 974 leads to SH2 domain-containing tyrosine phosphatase 2 (SHP2), also called protein tyrosine phosphatase, non-receptor type 11 (PTPN11) binding to these residues [48]. While binding of SHP2 to Tyr 986 leads to its activation it has been proposed that this is followed by SHP2-mediated dephosphorylation of Tyr 974, suggesting the ability of SHP2 to down-regulate activity of proteins binding at this site [48]. LEPR lacking Tyr 985 induces activation of ERK, although at a reduced rate, and this is due to an alternative ERK1/2 activation pathway, independent of receptor phosphorylation, *via* interaction of SHP2 and GRB2 with JAK2 [49]. JAK2 auto-phosphorylation at the Box1 motif leads to phosphorylation of insulin receptor substrate 1/2 (IRS1/2) which can activate the phosphatidylinositol 3-kinase (PI3K)/Akt pathway [47] (Figure 1).

Intriguingly, leptin retains the ability to stimulate STAT3 and ERK1/2 in cultured cells genetically deficient in JAK2 [50]. These JAK2-independent responses appear to be mediated by members of the Src family kinases (SFKs). Kinase inactive JAK2 introduced in JAK2-deficient cells enhances leptin signalling, suggesting that JAK2, besides its tyrosine kinase activity, can act as an adaptor to transduce leptin signals [50]. However, JAK2-dependent and-independent pathways seem to act cooperatively to mediate leptin responses [50].

Activation of STAT3 upon leptin binding also leads to activation of the negative feedback regulator suppressor of cytokine signalling 3 (SOCS3), which in turn attenuates leptin signalling by binding Tyr 985 and preventing JAK2 activation [51]. Resistance to leptin action is believed to be mediated through SOCS3 since *in vitro* overexpression of this protein blocked leptin-mediated signalling. In addition, another negative regulator of leptin signalling, and a possible contributor to its resistance, is protein tyrosine phosphatase 1B (PTP1B), a class 1 nonreceptor PTP. PTP1B is capable of inhibiting leptin signalling by dephosphorylating JAK2 [52].

## MOLECULAR EFFECTS OF LEPTIN ON PROSTATE CANCER

Long-term exposure to leptin was shown to enhance the growth of all three main prostate cancer cell lines (LNCaP, DU145 and PC-3) [53], where androgen-insensitive prostate cancer cell lines DU145 and PC-3 show a stronger proliferative response to leptin treatment in comparison to androgen-sensitive LNCaP cells [54]. Leptin also induces the expression of VEGF, transforming growth factor β1, and basic fibroblast growth factor in DU145 and PC-3 cells, stimulating cell survival pathways and leading to proliferation and angiogenesis [55], although these results have not been reproduced in another study [56]. Another mechanism of leptin-induced prostate cell proliferation was suggested in a recent study showing that leptin influences estrogen metabolism and causes an increase in the expression of estrogen receptor (ER)-α and a decrease in ER-β [57]. Leptin was shown to induce cellular migration of human prostate cancer mediated via upregulation of αvβ3 integrin and intracellular signal transduction [58].

*In vivo*, low-leptin *ob/ob* mice injected with murine androgen-insensitive prostate cancer cell line RM-1, developed larger tumours and had stronger Ki-67 staining than high-leptin *db/db* mice [59]. However, epidemiological evidence supports leptin influence on the promotion of prostate cancer [24] and developing larger tumours [18]. Leptin receptor mutation is significantly associated with an increased risk of prostate cancer [17]. Of note, new leptin receptor antagonists are currently becoming available for therapeutic targeting of obesity-associated pathways (reviewed in [60]). They already have been shown to inhibit *in vitro* and *in vivo* growth of breast cancer [61] and melanoma cells [62].

## CONCLUSIONS

It is now clearly established that obese patients with prostate cancer tend to have more aggressive disease, while there is still conflicting evidence regarding the association of obesity and risk of developing prostate cancer. Overall, the available evidence suggests that although it is unlikely for obesity to affect incidence of prostate cancer *per se*, obesity may affect the progression of existing prostate cancers, specifically the high-grade ones. The exact mechanism of this effect is unknown, and hypotheses range from the alteration in the testosterone effect on indolent *vs* high-grade tumours, to a multifactorial influence of adipose tissue secreted factors. Indeed, expression of multiple cytokine receptors including IL-6 and TNF-α is increased in prostate cancer in comparison to control tissue [63], and rises with Gleason grade [64]. Leptin is a prominent adipokine secreted by adipose tissue that has been linked to progression and metastasis of many cancers. Similarly to obesity, leptin was shown to correlate with increased prostate cancer risk and developing of larger tumours, while the data linking leptin levels with prostate cancer incidence is inconclusive. It is possible that obesity and leptin affect only specific types of prostate cancers or only certain events during tumour progression such as epithelial/mesenchymal transition, metastasis or angiogenesis. Therefore, further epidemiological studies should be combined with histopathological studies analysing adipokine and adipokine receptor expression in an attempt to classify different subtypes of prostate cancer that may be more prone to obesity-related changes. These could be further facilitated using metabolomics and systems biology approaches and such data could yield stronger associative evidence.

In a view of recent findings linking leptin signalling to the sphingolipid pathway it may be reasonable to investigate this link in prostate cancer, specifically in a view of developing new chemo- or radio-sensitizing therapies. This is particularly timely, as new leptin receptor antagonists are currently becoming available for therapeutic targeting. If there is a rationale for their use in prostate cancer, clear patient subsets need to be identified.
